# Nasal saline irrigation: prescribing habits and attitudes of physicians and pharmacists

**DOI:** 10.1080/02813432.2021.1880123

**Published:** 2021-02-11

**Authors:** Jesse Tapiala, Antti Hyvärinen, Sanna Toppila-Salmi, Eero Suihko, Elina Penttilä

**Affiliations:** a Department of Otorhinolaryngology – Head and Neck Surgery, Kuopio University Hospital and University of Eastern Finland, Kuopio, Finland; b Department of Otorhinolaryngology – Head and Neck Surgery, Kuopio University Hospital, Kuopio, Finland; c Skin and Allergy Hospital, Helsinki University Hospital and University of Helsinki, Helsinki, Finland; d Joensuun Uusi Apteekki and University of Eastern, Joensuu, Finland

**Keywords:** Nasal lavage, patient education, sinonasal symptoms, sinonasal conditions, adverse effects

## Abstract

**Objectives:**

To explore the opinions, the usage and the patient education given on nasal saline irrigation by physicians and pharmaceutical personnel working in Finland.

**Design:**

An internet-based survey with predetermined, multiple-choice answers.

**Setting:**

Primary care centres, occupational health centres and private care centres in Eastern Finland as well as pharmacies in Finland.

**Main outcome measures:**

Healthcare professionals views, practice and general knowledge of nasal irrigation for sinonasal symptoms and conditions.

**Results:**

We received 595 completed surveys (110 physicians, 485 pharmacists). The majority of the respondents recommended nasal saline irrigation for their patients either as a symptomatic treatment (98.0%) or to treat a specific condition (97.5%) such as acute rhinosinusitis, chronic rhinosinusitis and allergic rhinitis. Nasal saline irrigation was also often recommended as a prophylaxis for airway-infections (71.9%) and to enhance the health of the nasal mucosa (58.2%). In general, the possible adverse effects were recognised poorly by both professions. There was a clear difference between the two professions, as physicians were more conservative in recommending nasal saline irrigation and recognised possible adverse effects, such as epistaxis, pain, and dryness of the nose, better (75% vs. 59%, *p* = 0.002).

**Conclusions:**

Nasal saline irrigation seems to be a popular treatment recommended by many health care professionals in Finland. Physicians and pharmaceutical personnel had variable opinions on the indications, utility and risks of nasal saline irrigation. There are also clear differences between physicians and pharmaceutical personnel’s practices. There is a need to better educate professionals about nasal saline irrigation and to further study whether nasal saline irrigation is efficient and safe option for the different common sinonasal conditions.

**KEY POINTS**
- Little information is available on how physicians and pharmacists recommend nasal saline irrigation as a symptomatic treatment.
- Physicians and pharmacists seem to have variable opinions about the indications, utility and safety of nasal saline irrigation.
- The patient education given is in general very heterogenous.
- Both professions require more education to ensure that the usage remains as safe as possible for the patient.

## Introduction

The prevalence of acute rhinosinusitis (ARS) is about 6–15% in the Western world, chronic rhinosinusitis (CRS) is 10% and allergic rhinitis (AR) is about 25% [1–3]. The burden of sinonasal diseases is a global health and financial challenge. For example, in the US the annual direct costs for CRS are 8.6 billion dollars and rhinosinusitis is one of the top-ten most costly health conditions when both direct and indirect costs are taken into account [1]. Hence, cost-effective treatments are in high demand.

Many over-the-counter treatments claim to alleviate nasal symptoms, but their efficacy remains largely unproven [4]. In nasal saline irrigation (NSI) the patient rinses nasal cavities by instilling either isotonic or hypertonic saline solution into one nostril and allows it to drain out from the other nostril [5–8]. Some believe it is an efficient, cheap and safe alternative treatment [9]. However, the grade of evidence for its effectiveness is of low quality based on Cochrane reviews and the reviews conclude that no recommendations can be made [6–8] (Table 1). Still, it is often recommended in different guidelines (Table 2). It is also still unclear whether isotonic or hypertonic solutions should be favoured [6–8]. NSI is also used in postoperative care to promote mucosal recovery [18,19]. The evidence is limited and no official guidelines exist [18,19].

**Table 1.** Recommendations from Cochrane Reviews considering the usage of nasal saline irrigation for different sinonasal diseases and the quality of evidence.

Indication	Recommendation	Quality of evidence
Acute rhinosinusitis and common cold [6]	May be beneficial, no recommendation can be made	Low or very low
Allergic rhinitis [8]	May be beneficial, no recommendation can be made	Low or very low
Chronic sinusitis [7]	May be beneficial, no recommendation can be made	Low or very low
Atrophic rhinitis [10]	No recommendation can be made	
Asthma	None exist	
Post-operative care after functional endoscopic sinus surgery	None exist	
Regular cleaning after functional endoscopic sinus surgery	None exist	

**Table 2.** Recommendations from International Guidelines and Finnish National Guidelines considering the usage of nasal saline irrigation (NSI) for different sinonasal diseases and the strength of recommendation (if reported).

Indication	Recommendation	Strength of recommendation
Acute rhinosinusitis and common cold (ARS)		
EPOS 2020 for adults and children [1]	NSI possibly has benefits for symptom relief mainly in children	Ib^#^
EPOS 2020 for adults with acute post-viral rhinosinusitis [1]	No recommendation can be given based on very low quality of evidence	
EPOS 2020 for adults with acute bacterial rhinosinusitis [1]	No recommendation can be given based on very low quality of evidence	
Finnish Guideline: ARS and common cold [11]	No mention of NSI	
Allergic rhinitis (AR)		
ARIA 2008 [3]	Recommended in a pharmacy setting	
Finnish guideline: AR [12]	No mention of NSI	
AAFP Clinical Practice Guideline: AR 2015 [13]	No mention of NSI	
Asthma		
Global initiative for asthma (GINA) [14]	No mention of NSI	
Finnish guideline: Asthma [15]	No mention of NSI	
International ERS/ATS guideline for asthma [16]	No mention of NSI	
Atrophic rhinitis		
Finnish guideline: Atrophic rhinitis [17]	Recommended	
Chronic sinusitis (CRS)		
EPOS 2020 for adults with chronic rhinosinusitis [1]	Recommended	Ia*
EPOS 2020 for children with chronic rhinosinusitis [1]	Recommended	Ib+^#^
Finnish guideline: CRS [11]*	Recommended	Low
Post-operative care after functional endoscopic sinus surgery (FESS)		
No official guidelines exist		
Regular cleaning after FESS		
No official guidelines exist		

*Note.* EPOS 2020 reports the level of evidence instead of strength of recommendation.

*Based on older Cochrane reviews, *Ia: based on systematic review of RCTs; #Ib: based on individual RCT.

Only a few studies have been made to evaluate the use of NSI by healthcare professionals [9,20], but based on those it seems to be quite popular. There is little evidence of how patients should be educated on its safe usage and by who [1,3,6–8,11]. This can lead to undesired and even potentially harmful treatments such as using hypotonic solutions for irrigation, even though they have been shown to cause damage to the respiratory epithelium [21].

We aimed to explore the opinions, the usage and the patient education given on NSI by physicians and pharmaceutical personnel working in Finland. Our two main hypotheses were that there would be significant differences between the two professions included in our survey and that the results would be highly variable.

## Materials and methods

### Study design

We conducted a cross-sectional survey regarding the usage of NSI (not including nasal salt sprays) by pharmacists and physicians in Finland. The survey was carried out between 7 February 2019 and 18 March 2019 using an internet-based survey platform, Surveypal. The survey was distributed nationwide for pharmaceutical personnel, but more locally for the physicians as we had no means to achieve nationwide coverage. The anonymous survey was open for six weeks. No ethical approvals were required for this study.

### Setting and population

Pharmaceutical personnel were approached with an email containing the link to the survey as well as a short cover letter. The link was included in a regular newsletter sent to all members of the Association of Finnish Pharmacies and published on their official website to eliminate the possibility of selection bias.

Physicians were recruited from a few of the largest private care centres, primary care centres as well as occupational health centres in Eastern Finland. Our target group consisted of primary care physicians, occupational health physicians and private care physicians, including general practitioners and specialists. If the invite was accepted, it was generally distributed to all the working staff by internal email containing the cover letter.

### Questionnaire design

There is no validated survey available, so we had to develop our own. We settled for 19 multiple-choice questions. The questionnaire (Appendix 1) was reviewed and commented by a small group of physicians and pharmacist before finalizing it and translating it to an internet-based survey system.

### Statistical analysis

After closing the survey, we used the automatic tool from Surveypal to export the data. Every responder was automatically given a unique, anonymous ID-number. The authors explored the findings in relation to the goals of the study and identified those worth of pursuing more closely. These findings were then selected for statistical analysis, which was carried out with the SPSS Base 25.0 Statistical Software Package (SPSS Inc, Chicago, IL, USA). Descriptive statistics and proportions for categorical variables were calculated. Fisher’s exact test was used to determine whether there was a difference in administering NSI between physicians and pharmacists as well as other subgroups and categories. The continuous variables were described as median values and interquartile ranges (IQR). Differences between the two groups for continuous variables were calculated with independent samples Mann-Whitney U test. Two-tailed *p*-values of <0.05 were considered statistically significant.

## Results

We received 595 completed surveys, most of which (485; 81.5%) were from pharmacy personnel. The remaining 110 (18.5%) answers came from physicians. A total of 855 people opened the link leading to the survey (a response rate of 69.6%). We managed to get answers from 5.6% (485/8862) of all the pharmacy personnel working in Finland [22]. Because of the method for distributing the survey, we have no data about how many physicians we managed to contact and were unable to estimate the response rate to the survey. Reported physician subspecialties were general practice 33.6% (37/110), occupational health 47.3% (52/110) and private care 17.3% (19/110). Most respondents had over 10 years of work experience (384; 64.9%). Our sample was well represented from a geographical standpoint and in terms of the working environment of the respondents (58.7% in cities, 41.3% in population centres). There were no significant differences when comparing the answers in terms of working experience (less than 5 years of experience vs. more than 5 years of experience). Due to the small sample size of physician’s subspecialties, it was not feasible to examine possible differences between them.

### Indications for NSI

**Figure 1.**
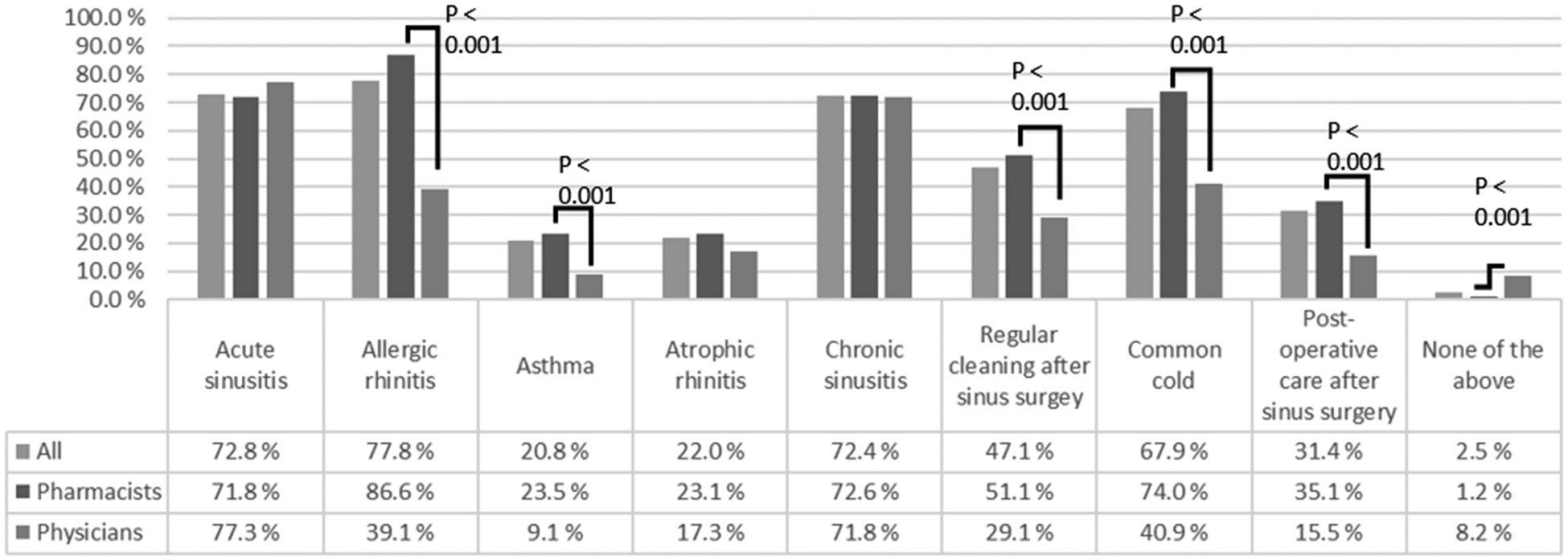
Conditions that nasal saline irrigation is recommended for by Finnish physicians (*n* = 110) and pharmacists (*n* = 485).

Most of the respondents recommended NSI for ARS, CRS, AR and the common cold (Figure 1). NSI was also administered for asthma and atrophic rhinitis and as post-operative care for functional endoscopic sinus surgery. Only a small minority did not recommend NSI for any of these conditions. Pharmacist recommended NSI significantly more for different conditions such as AR, asthma and the common cold (Figure 1).

**Figure 2.**
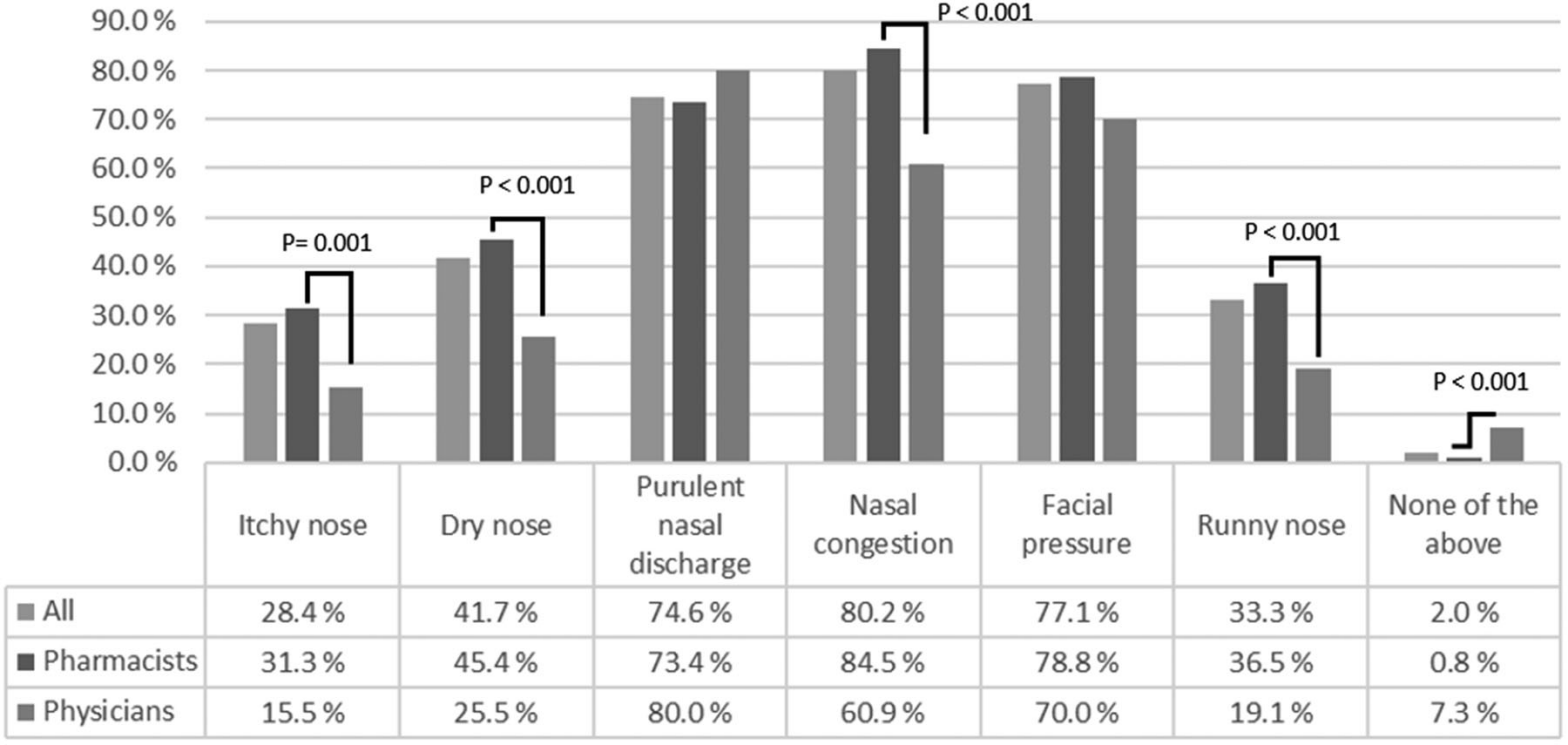
Symptoms that nasal saline irrigation is recommended for by Finnish physicians (*n* = 110) and pharmacists (*n* = 485).

The sinonasal symptoms for which NSI was most commonly administered were nasal congestion, facial pressure and purulent nasal discharge (Figure 2). Irrigations were a slightly less popular choice for dryness of the nose, runny nose and nasal irritation. Only a small minority of the respondents did not recommend NSI for any of the previous symptoms. Pharmacist recommended NSI significantly more for different symptoms such as itching of the nose, dryness of the nose, nasal congestion, and runny nose.

NSI was also often recommended for the prevention of upper respiratory infections (*n* = 428; 71.9%) and to enhance the well-being of the nasal mucosa (*n* = 346; 58.2%). Pharmacist recommended NSI more frequently for the prevention of infections (77.9% vs. 45.5%, *p* < 0.001) and for the well-being of the nasal mucosa (64.1% vs. 31.8%, *p* < 0.001).

### Safety and popularity

**Figure 3.**
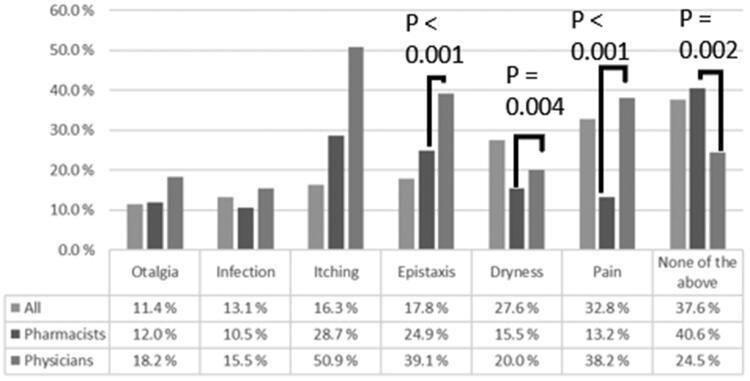
Possible adverse effects of nasal saline irrigation as reported by physicians (*n* = 110) and pharmacists (*n* = 485) working in Finland.

The most commonly recognised adverse effects can be seen in Figure 3. In general, pharmacists recognised fewer possible adverse effects.

53.7% (*n* = 320) of the respondents estimated recommending irrigations for 1–20% of their patients with any nasal symptoms and 43.7% (*n* = 260) for 21–100% of the patients, while only 2.5% (*n* = 15) did not recommend irrigations at all.

### Means of irrigation and patient education

In summary, the most popular method for NSI was a Neti pot (*n* = 477; 80.7%) filled with an isotonic solution (*n* = 440; 73.9%) that was 30–40 degrees Celsius (*n* = 417; 70.1%). Hypertonic (*n* = 18; 3.0%) or hypotonic (*n* = 11; 1.8%) solutions were rarely administered and the rest did not instruct the tonicity (*n* = 126; 21.2%). Volumes of 51–100 ml (*n* = 72; 12.2%) and 101–200 ml (*n* = 83; 14.0%) were recommended the most, while the majority gave no recommendation at all (*n* = 316; 53.4%). Many of the respondents recommended homemade solutions (*n* = 483; 81.2%) as opposed to purchasing ready-to-use nasal solutions from pharmacies (*n* = 325; 54.6%). Irrigations were most instructed as a daily (*n* = 222; 37.8%) or twice-a-day (*n* = 277; 47.2%) therapy. Regular irrigations were recommended by 77 (12.9%) of the respondents, while the rest (*n* = 518; 87.1%) opted for a short-term usage (mean: 6.52 days, SD = 3.9, min = 1 day and max = 30 days). There was no significant difference in the length of the recommended use between pharmacists and physicians.

Cleaning of the device used for irrigations was commonly instructed to be done once before or after use (*n* = 356; 59.8%). Cleaning before and after use was recommended by 13.9% (*n* = 83) of the respondents, while 21.5% (*n* = 128) recommended cleaning once a week and 4.7% (*n* = 28) even less frequently. The most popular cleaning methods were rinsing with running water (*n* = 272; 45.7%) or cleaning with boiling water (*n* = 214; 36.0%). Physicians were much more likely to not give instructions regarding the desired volume (51.3% vs. 60.9%, *p* = 0.073), tonicity (17.3% vs. 38.1%, *p* < 0.001), temperature (5.4% vs. 22.7%, *p* < 0.001) and cleaning (18.6% vs. 51.8%, *p* < 0.001) compared to pharmacists.

## Discussion

This study was to evaluate how physicians and pharmaceutical personnel recommend NSI to patients in Finland. Nasal saline irrigation was recommended by almost all of the respondents either as a symptomatic treatment or for a specific condition. Possible adverse effects were recognised poorly. Our findings seem to be in line with the two other studies exploring the usage of NSI by physicians, as they found out that 99.3% (854/860) or 87% (286/330) of physicians use NSI either as a standalone or adjunctive therapy [9,20]. The study by Marchisio *et al* surveyed the use of NSI by primary health care pediatricians working in Northern Italy, while the study by Rabago *et al* investigated the use by family physicians in Wisconsin, USA. Both studies focused on the usage of NSI for upper respiratory infections [9,20].

To our knowledge, this is the first study to show a clear difference between the two professions in recommending NSI usage for patients: physicians were more conservative in recommending NSI and recognised possible adverse effects better. This difference could be due to physicians knowing the patient’s general diseases better, putative risks, or physicians’ lack of knowledge related to the use of NSI.

In this study, almost all the respondents recommended NSI as a symptomatic treatment for their patients. It was most frequently administered for nasal congestion, facial pressure and purulent nasal discharge. In general, pharmacy personnel seemed to recommend NSI significantly more frequently for symptomatic treatment than physicians did. Pharmacy personnel also recommended NSI more readily. This difference could result from the fact that many of these symptoms and disorders are mild and thus people are more likely to turn to pharmacies for over-the-counter treatment for their symptoms, rather than to visit a doctor. Another explanation could be that physicians have less pressure to offer an instant solution to a patient’s problem and can more easily adopt a wait-and-see approach and on the other hand, they also have alternative management options, including those not available over-the-counter. It is also possible that marketing could play some role.

NSI has a low or very low level of evidence in the treatment of ARS, CRS, AR and the common cold [6–8]. Interestingly, our study showed that NSI was also frequently recommended for the prevention of upper airway infections and the well-being of the nasal mucosa. Almost a fifth of the responders also recommended NSI for asthma, even though it is not included in any of the guidelines [14]. We could not find any studies that have investigated these indications. Physicians were significantly less likely to recommend irrigations for these reasons. It remains unclear whether these might be a sole reason for recommending NSI to an asymptomatic patient or just a conceived beneficial effect on top of the intended symptomatic treatment.

There is very little information available about the frequency and the scope of the possible adverse effects in the literature. Most of the studies included in the Cochrane reviews had inadequate reporting or collecting of the possible adverse effects [6–8]. However, dryness of the nose, pain, irritation and epistaxis were mentioned [6–8]. Similarly, a significant portion of our responders thought that NSI carries no negative side-effects. However, the safety of prolonged and regular, multiple times a day usage is unclear.

At the same time, increasing antimicrobial resistance is an ever-present global threat [23]. A recent quality assessment study in Denmark showed that almost 65% of the patients diagnosed with ARS received antimicrobial treatment in a general practice setting [24]. The evidence-based acceptable range is between five to ten per cent – indicating a significant overuse of antibiotics [25]. This phenomenon is well recorded in other studies as well [26–29]. A vast majority of the patients with ARS believe that healing requires medication and one of the reasons for the overprescribing is the pressure clinicians feel to meet the expectations of their patients [30,31]. We believe this could be one of the reasons behind the observed popularity of NSI in our study, even though it is not an evidence-based treatment.

Despite the reported adverse events of NSI, we found that NSI is recommended to a relatively wide range of upper airway symptoms, at least in Finland. Moreover, we found that healthcare and/or pharmaceutical professionals give relatively little and very variable instructions on how to use and take care of the NSI device. We identified significant differences between the two professions that can probably be explained by the different education basis, as physicians’ practices are generally much more in line with the current guidelines when compared to the pharmaceutical personnel. Hence, it might be important to better deal practical summary of evidence-based consensus of NSI’s indications, its potential risks, and its use, to all healthcare and pharmaceutical professionals who work with people having upper airway problems. There is also a need to study the efficacy and safety of NSI further in different upper airway conditions. There has not been a single study of how the patients use NSI in real life. Patients deserve good education by professionals as upper airway symptoms are common and patients seek for symptom relief by using over-the-counter management. Moreover, not all patients can follow the written instructions provided by the device. An option could thus be to provide publicly available instructions of how to use NSI by healthcare professionals, such as this instructional video available at the official website produced jointly by the Finnish University Hospitals [32]. It is unclear how many of the professionals know that a video like this exists and how many recommend it to their patients.

Our study has some limitations. Selection bias may have occurred as the survey was distributed for pharmacists with national-level coverage while physician recruitment was restricted to the Eastern Finland region. Secondly, the study design prevented us from calculating the exact responder rate or performing a non-responder analysis. It could be thus possible that those professionals who responded had more positive opinions towards NSI than the non-responders. Thirdly, there is a clear bias towards pharmacists as a clear majority of the responders were pharmacists. This survey was to evaluate the opinions in general and thus we were not able to get data of the frequency and methods of how professionals educate patients to use NSI in their everyday practice. It is also possible that our findings do not represent the actual practise or opinions. We acknowledge that the responding physicians represent only a small portion (3.7%) of the licenced physicians in Eastern Finland [33] and thus our results may not fully correspond to the general opinions of all physicians. However, we believe our study design was relevant when considering the goal of our study and that we managed to reach those goals.

In conclusion, NSI is often recommended to patients by healthcare or pharmaceutical professionals in Finland. We demonstrated that pharmaceutical professionals recognize less the potential harm of using NSI and they recommend NSI for a wider range of sinonasal conditions, including those that have not currently been included into the evidence-based recommendations in the guidelines. Also, there is heterogeneity in giving instructions on how to perform NSI and how to take care of the device. There is a need to improve subjects’ as well as healthcare/pharmaceutical professionals’ knowledgé of NSI. This could be achieved by open access instructions and videos, but most importantly, they need to be promoted more vigorously to bring them to the attention of the professionals. There is also a need to study more the efficacy of NSI in various sinonasal conditions. As of now, the methods used are very diverse, sometimes even potentially harmful (i.e. using hypotonic solutions) and for some questionable indications.

## Appendix 1

Translated survey for Finnish physicians and pharmacists concerning the usage, patient education and knowledge of nasal saline irrigations (NSI). Questions 2a and 2b were only asked if the responder was not a physician. Question 10a was asked only if the responder opted for short-term usage of NSI instead of regular use. This survey was conducted with Surveypal. We aimed for a balance between getting enough information and making the survey as short as possible to ensure better response rate. To make the data more easily manageable, we opted mainly for pre-determined answers in the form of multiple-choice-questions.

1. What is your work experience? (select one)
  - 0–2 years
  - 2–5 years
  - 5–10 years
  - 10–15 years
  - 15–20 years
  - Over 20 years
2. What is your occupation? (select one)
  - Head pharmacist
  - Pharmacist
  - Provisor
  - Other pharmacy personnel
  - Primary care physician
  - Occupational physician
  - Private practioner
2a. In what province is your pharmacy located? (select one)
  - Uusimaa
  - Varsinais-Suomi
  - Satakunta
  - Kanta-Häme
  - Pirkanmaa
  - Päijät-Häme
  - Kymenlaakso
  - Etelä-Karjala
  - Etelä-Savo
  - Pohjois-Savo
  - Pohjois-Karjala
  - Keski-Suomi
  - Etelä-Pohjanmaa
  - Pohjanmaa
  - Keski-Pohjanmaa
  - Pohjois-Pohjanmaa
  - Kainuu
  - Lappi
  - Ahvenanmaa
2b. Is your pharmacy located in a city or in a population centre? (select one)
  - In a city
  - In a population centre
3. Would you recommend nasal saline irrigations (NSI) for the following age groups? (you can choose multiple)
  - <1-year-olds
  - 1–7-year-olds
  - 8–18-year-olds
  - 18–65-year-olds
  - >65-year-olds
  - All of the above
4. Would you recommend NSI for a completely healthy individual? (select one)
  - Yes
  - No
5. Would you recommend NSI for prophylaxis of upper airway infections? (select one)
  - Yes
  - No
6. Would you recommend NSI for the betterment of the well-being of the nasal mucosa? (select one)
  - Yes
  - No
7. For which of the following symptoms would you recommend NSI for? (you can choose multiple)
  - Itching of the nose
  - Dryness of the nose
  - Purulent nasal discharge
  - Nasal congestion
  - Facial pressure
  - Runny nose
  - None of the above
8. For which of the following conditions would you recommend NSI for? (you can choose multiple)
  - Acute rhinosinusitis
  - Allergic rhinitis
  - Asthma [Database]
  - Atrophic rhinitis
  - Chronic rhinosinusitis
  - Regular cleaning of the sinuses after sinus surgery
  - Common cold
  - Post-operative treatment after sinus surgery
  - None of the above
9. Estimate how often do you recommend NSI for a patient with nasal symptoms (select one)
  - 0%
  - 1–20%
  - 21–40%
  - 41–60%
  - 61–80%
  - 81–100%
10. Would you recommend NSI to be used regularly or more as a short-term solution? (select one)
  - Short-term
  - Regularly
10a. The recommended length of use on average? (open field)
11. How often would you recommend NSI to be administered? (select one)
  - Once a week
  - Every few days
  - Once a day
  - Twice a day
  - More than twice a day
12. What mean for NSI would you primarily recommend? (select one)
  - Neti pot
  - Physiomer Normal Jet & Spray or Strong Jet
  - Syringe
13. What should the volume of the solution used be? (select one)
  - <20 ml
  - 20–50 ml
  - 51–100 ml
  - 101–200 ml
  - 201–500 ml
  - >500 ml
  - I don’t give instructions concerning the volume
14. What should the tonicity of the solution used be? (select one)
  - Hypotonic (<0.9% NaCl)
  - Isotonic (0.9% NaCl)
  - Hypertonic (>0.9% NaCl)
  - I don’t give instructions concerning the tonicity
15. What should the temperature of the solution used be? (select one)
  - 10–20° Celsius
  - 20–30° Celsius
  - 30–40° Celsius
  - I don’t give instructions concerning the temperature
16. What kind of solutions would you recommend for NSI? (you can choose multiple)
  - Sachet bought from pharmacy or elsewhere
  - Ready-to-use saline from pharmacy or elsewhere
  - Homemade solution
17. How would you recommend cleaning of the device used for NSI? (you can choose multiple)
  - Rinsing with running water
  - Cleaning in a microwave oven
  - Cleaning with a antimicrobial solution
  - Cleaning with boiling water
  - Cleaning with soap
  - I don’t give instructions concerning the cleaning
18. How often would you recommend cleaning of the device used for NSI? (select one)
  - Few times a month
  - Weekly
  - Every time before or after use
  - Every time before and after use
19. What adverse effects NSI could possibly cause? (you can choose multiple)
  - Otalgia
  - Infections
  - Itching
  - Epistaxis
  - Dryness
  - Pain
  - None of the above
